# Acute Ischemic Stroke Post Honeybee Sting: A Rare Case Report

**DOI:** 10.7759/cureus.31851

**Published:** 2022-11-24

**Authors:** Ruchita Kabra, Amol Andhale, Sourya Acharya, Sunil Kumar, Rucha Sawant

**Affiliations:** 1 Department of Internal Medicine, Jawaharlal Nehru Medical College, Datta Meghe Institute of Medical Sciences, Wardha, IND; 2 Department of Medicine, Jawaharlal Nehru Medical College, Datta Meghe Institute of Medical Sciences, Wardha, IND

**Keywords:** unusual reaction, neurological, anaphylaxis, ischemic stroke, bee sting

## Abstract

Stings by bees or wasps are frequent worldwide. From minor urticaria to severe anaphylaxis, allergic symptoms are typically present. Various reports of unexpected reactions to bee stings affecting the neurological, renal, cardiac, pulmonary, and ocular systems have been published. Here, we present a rare instance of subacute bee sting syndrome that resulted in an ischemic stroke over a 24-hour period.

## Introduction

The second leading cause of death and a significant contributor to disability worldwide is stroke [1]. Besides anaphylactic allergic reactions, hemorrhagic and ischemic strokes can also be led by the venom of honeybee’s sting (Apis mellifera, order Hymenoptera). These neurological impairments are caused either directly by the toxins released by venom itself or indirectly by anaphylactic shock or a catabolic response to pain. Proteins, amines, and enzymes with biological activity are present in venom. Due to local, regional, or systemic allergic reactions, these chemicals directly or indirectly result in a wide range of medical complications [1]. Localized swelling and edema are hallmarks of the local reactions, which often go away within 24 hours. Regional and systemic reactions, on the other hand, significantly increase morbidity and mortality. These reactions can appear in various ways, ranging from cerebral infarctions and intracranial hemorrhage to allergy, hypotension, rhabdomyolysis, seizures, and disseminated intravascular coagulation (DIC) [2]. We report a case of infarct in left corona radiate occurring within five to six hours of a bee sting in a 49-year-old man presenting with seizure.

## Case presentation

**Figure 1:**
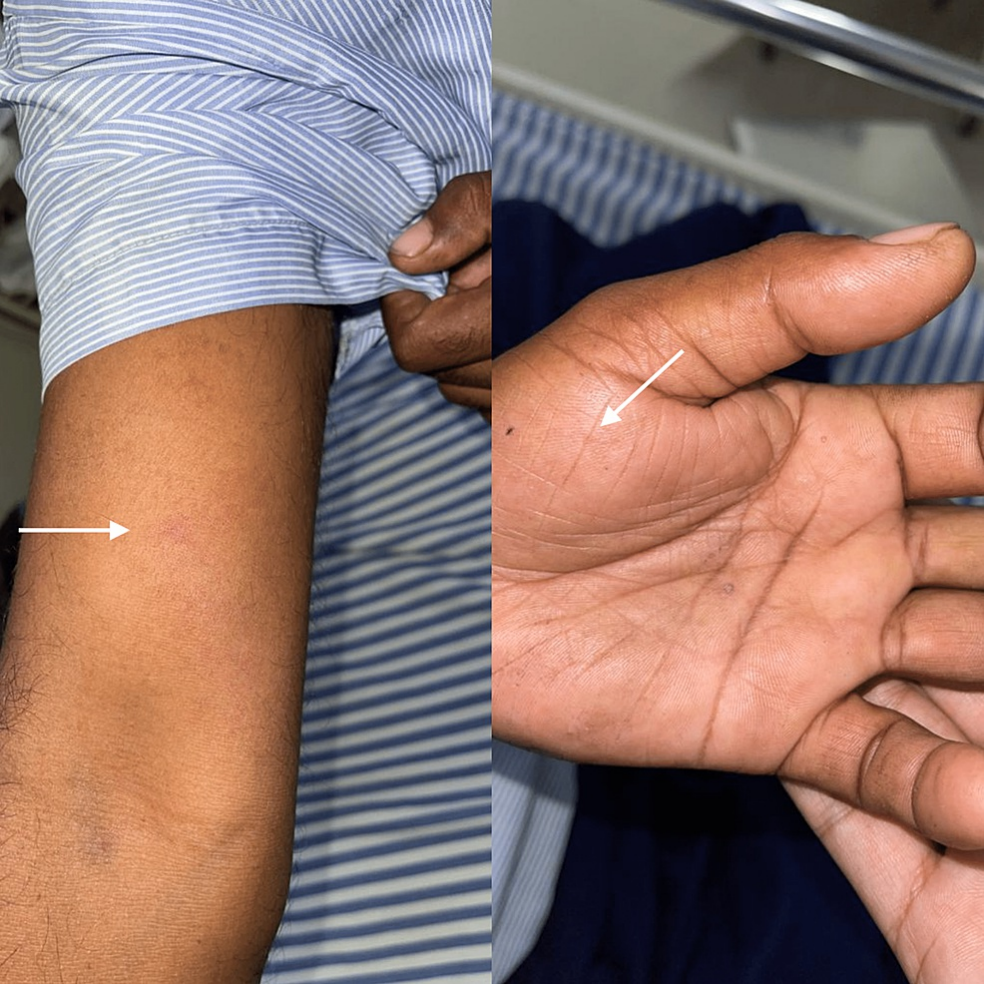
Bee sting marks on the right arm (left) and left palm (right) (white arrow).

A 49-year-old male, farmer by occupation, was bitten by a honeybee over his hand and arms while working in his fields. He developed swelling over the right arm and left palm at the bite site, which was painful and tender (Figure 1).

He had no history of fever, cough, cold, nausea, vomiting, breathlessness, chest pain, palpitations, blackouts, or giddiness. He had no history of smoking or alcohol. On admission, his vitals were as follows: Glasgow Coma Score of 14/15 (E4V5M5), heart rate of 86 beats per minute, BP of 130/80 mmHg, and respiratory rate of 22 breaths per minute. The electrocardiogram showed normal rate and rhythm.

**Figure 2:**
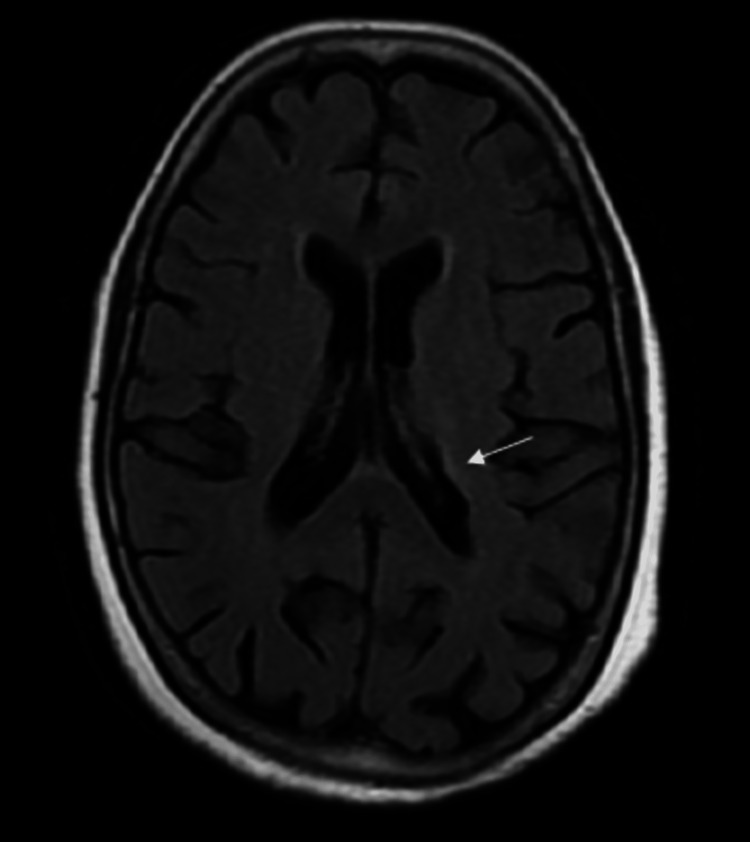
T2 FLAIR image of brain MRI showing hyperintense signal in the left corona radiata (white arrow). MRI, magnetic resonance imaging; FLAIR, fluid-attenuated inversion recovery

**Figure 3:**
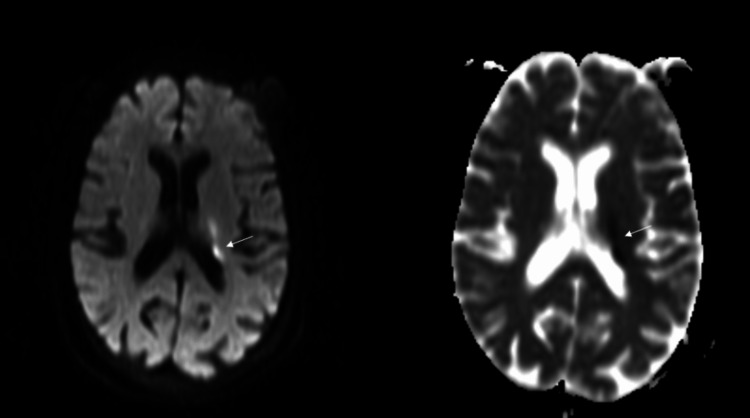
DWI showing diffusion restriction in the left corona radiata (white arrow on left) with corresponding low signal on ADC (white arrow on right). ADC, apparent diffusion coefficient; DWI, diffusion-weighted imaging

**Figure 4:**
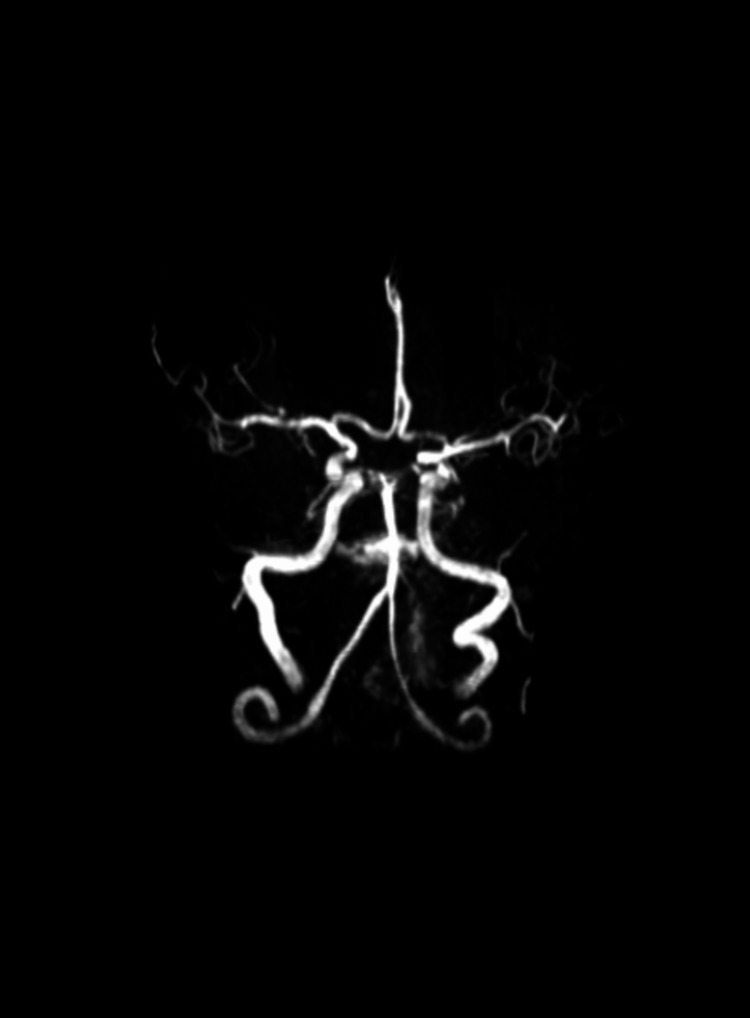
MR angiography showing normal arteries. MR, magnetic resonance

He was managed with antihistamines and steroids and was admitted to the emergency department of Acharya Vinoba Bhave Rural Hospital, Wardha, Maharashtra, India, for observation. After five to six hours of the sting, he had an episode of a generalized tonic-clonic seizure followed by right hemiparesis. He was started on anti-epileptic drugs. Neuroimaging was performed. T2 fluid-attenuated inversion recovery (FLAIR) brain MRI showed hyperintensities in the left corona radiata (Figure 2). Diffusion-weighted imaging (DWI) showed diffusion restriction to corresponding low signals on the apparent diffusion coefficient (ADC; Figure 3). Magnetic resonance (MR) angiography was normal (Figure 4). He was shifted to the medical intensive care unit.

He had no history of fever, cough, cold, nausea, vomiting, breathlessness, chest pain, palpitations, blackouts, or giddiness. He had no history of smoking or alcohol intake. There was no similar episode in the past or any significant history, including surgical history. Also, there was no similar episode in his family.

On examination, his vitals were as follows: Glasgow Coma Score of 9/15 (E2V3M4), heart rate of 90 beats per minute, BP of 132/86 mmHg, and respiratory rate of 24 breaths per minute. The cardiovascular system examination was normal, respiratory system auscultation showed normal air entry on both sides, and neurological examination showed sluggishly reactive pupils and plantar reflex extensor on the right side and flexor on the left side.

His blood reports suggest hemoglobin 13.2 g/dL, total leukocyte counts 10,220 mm^-3^, and platelet counts 2.5 lakhs/μL. Kidney function tests showed normal values like urea 32 mg/dL and serum creatinine 0.9 mg/dL. Liver function test was within normal limits such as alanine aminotransferase (ALT) 38 U/L, aspartate aminotransferase (AST) 72 U/L, alkaline phosphatase 166 U/L, serum albumin 3.1 gm/dL, and total serum bilirubin 1.3 mg/dL. Serum calcium 7.6 mg/dL, serum magnesium 2.6 mg/dL, serum sodium 136 mEq/L, and serum potassium 4.8 mEq/L. The serum fibrinogen level was 380 mg/dL. The coagulation profile was normal, with prothrombin time (PT) of 13.2 seconds and activated partial thromboplastin time (APTT) of 32.4 seconds, and the international normalized ratio (INR) was 1.2. The Carotid Doppler study revealed normal results with no abnormality.

He was treated with injectable mannitol, antiplatelet, antibiotics, antiepileptics, and other supportive management and hydration. Limb physiotherapy was provided. Repeat imaging showed no increase in the infarct size or any hemorrhagic transformation. The patient was continued on antiplatelet aspirin 75 mg once a day and anticonvulsant levetiracetam 500 mg twice a day. Physiotherapy was continued for rapid recovery. On follow-up after one month, he showed improvement symptomatically. After six months, he recovered neurologically and returned to his activities.

## Discussion

There is an increase in cytokines following bee stings, particularly interleukins such as IL-1, IL-6, and IL-8 and tumor necrosis factor (TNF) [3], which has negative consequences on the immunological, cardiovascular, central neurological, and skeletal muscle systems. In our case, acute ischemic stroke was developed.

Acute strokes have a complicated and multivariate pathogenesis that includes vascular phenomena of embolic type, hypotension, hypoxemia, and malignant hypertension with hemorrhage, resulting in arrhythmias, hypercoagulability, and immunological hyperreactivity. Vasoactive agents found in bee venoms such as histamine, leukotrienes, thromboxane, and serotonin can cause constriction of vessels, which can be exacerbated by exogenous epinephrine and platelet aggregation. Indirect harmful effects of bee venom include hyaluronidase, phospholipase 2 (PLA2) enzymes, and melittin toxin, which contains polypeptides that harm the cell membranes. The components of honeybee venom, particularly melittin, which blocks complement cleavage and bradykinin (BK) release, can cause hemorrhage [4,5]. The phospholipase enzyme is the most lethal component of bee venom. However, melittin and phospholipase work together to produce a more substantial hemolytic effect, which causes excessive and unexpected bleeding and hemorrhagic stroke.

The neurological symptoms caused by bee venom include cavernous sinus thrombosis, polyradiculopathy, cranial nerve palsies, stroke, and epilepsy. With a median of 16 hours, the reported time between envenomation and the stroke ranged from 15 minutes to 4 days [2]. Five to six hours after being envenomated, our patient first experienced a stroke. In our case, it was doubtful whether the stroke was caused by the activation of the cervical ganglia (sympathetic ganglia), anaphylactic shock, or hypotension because there was no evidence of hypotension or an allergic reaction. The patient had only been stung on his hand.

In our case, the coagulation profile of APTT and PT was normal. According to Petroianu et al., venom PLA2 is known to lead to aberrant coagulation. They claimed that high PLA2 content in blood had an impact on several coagulation markers. High PLA2 content in blood was reported to be correlated with the prolongation of PT, APTT, and antithrombin III [6]. Leukotrienes and thromboxane are also present in bee venom, which cause cerebral infarction by contributing to vasoconstriction. The formation of a prothrombotic condition is triggered by the activation of mast cells by bee venom [3]. Therefore, in our case, it is probable that the harm to the liver cells was caused by the venom's induction of a reversible prothrombotic condition. Therefore, hepatic dysfunction also had a role in the loss of consciousness that eventually led to a coma.

Various conditions are rare and associated with acute ischemic strokes like organophosphorus poisoning leading to the intermediate syndrome, suicidal method of hanging, or infection such as COVID-19 [7-9].

Corticoids, antihistamines, and antiepileptic medications should all be part of the treatment plan to reduce allergic reactions and control seizures. Rapid recognition and a logical strategy are preferable in cases of serious consequences such as brain infarctions, intracranial hemorrhages, and strokes.

## Conclusions

Strokes and other neurological conditions linked to bee venom are extremely uncommon. In such situations, if the presenting symptoms are severe, there is a chance of an ischemic stroke, which may consist of generalized tonic-clonic seizures. Such situations ought to be handled carefully and without further delay.
